# Global Research Mapping of Psycho-Oncology Between 1980 and 2021: A Bibliometric Analysis

**DOI:** 10.3389/fpsyg.2022.947669

**Published:** 2022-07-13

**Authors:** Tauseef Ahmad, Eric David B. Ornos, Shabir Ahmad, Rolina Kamal Al-Wassia, Iqra Mushtaque, S. Mudasser Shah, Basem Al-Omari, Mukhtiar Baig, Kun Tang

**Affiliations:** ^1^Vanke School of Public Health, Tsinghua University, Beijing, China; ^2^Department of Epidemiology and Health Statistics, School of Public Health, Southeast University, Nanjing, China; ^3^College of Medicine, University of the Philippines Manila, Manila, Philippines; ^4^Department of Agriculture, Bacha Khan University Charsadda, Charsadda, Pakistan; ^5^Department of Radiology, Radiation Oncology Unit, King Abdulaziz University Hospital, Jeddah, Saudi Arabia; ^6^Department of Psychology, Bahauddin Zakariya University, Multan, Pakistan; ^7^Department of Psychosomatics and Psychiatry, School of Medicine, Zhongda Hospital, Southeast University, Nanjing, China; ^8^Department of Epidemiology and Population Health, College of Medicine and Health Sciences, Khalifa University, Abu Dhabi, United Arab Emirates; ^9^KU Research and Data Intelligence Support Center (RDISC), Khalifa University of Science and Technology, Abu Dhabi, United Arab Emirates; ^10^Department of Clinical Biochemistry, Faculty of Medicine, Rabigh, King Abdulaziz University, Jeddah, Saudi Arabia

**Keywords:** psycho-oncology, bibliometric study, Scopus^®^ database, Bradford’s law, Lotka’s law

## Abstract

**Background and Aim:**

Psycho-oncology is a cross-disciplinary and collaborative sub-specialty of oncology that focuses on the psychological, behavioral, ethical, and social aspects of cancer in clinical settings. The aim of this bibliometric study was to analyze and characterize the research productivity and trends in psycho-oncology between 1980 and 2021.

**Methodology:**

In May 2022, the Scopus^®^ database was searched for psycho-oncology-related publications using predetermined search keywords with specific restrictions. Lotka’s law was applied to check the authors’ productivity, while Bradford’s law was used to assess the core journals in this field. The data was analyzed for different bibliometric indicators in the Biblioshiny package, an RStudio tool for bibliometric analysis.

**Results:**

The initial search resulted in a total of 2,906 publications. Of which, 1,832 publications were included in the final analysis, published between 1980 and 2021. The analyzed publications were written by 7,363 authors from 74 countries and published in 490 journals. There has been a significant increase in psycho-oncology-related publications after 2010. The most productive year was 2021 (*n* = 365). The annual scientific growth rate was found to be 13.9%. The most relevant leading author in terms of publications was Luigi Grassi from the University of Ferrara, Italy (*n* = 42). Lotka’s law found that the number of authors declined as the number of papers written increased. The core journals were Psycho-Oncology, Supportive Care in Cancer, and Journal of Psychosocial Oncology. The most frequently used author’s keywords other than searching keywords were cancer, oncology, quality of life, depression, and anxiety. Recent psycho-oncology-related topics included mental health, COVID-19 infection in humans, people, pandemic, and tumor. The University of Sydney was the top-ranked institution. The leading country in terms of publications, citations, corresponding author country, and international collaboration was the United States of America (United States). The United States had the strongest collaboration with Australia and Canada.

**Conclusion:**

The research hotspots include mental health conditions and interventions in cancer patients. We identified international collaboration and research expenditure to be strongly associated with psycho-oncology research productivity. Researchers’ collaboration, which is visible among developed countries, should be extended to low-income countries in order to expand psycho-oncology-related research and understanding.

## Introduction

The treatment and diagnosis of cancer is the primary life stressor for patients, their families, and partners, and it is linked with psychosocial and physical difficulties that can develop at any point during the disease and continue throughout life (Zabora et al., 2001; Pitceathly and Maguire, 2003; Chambers et al., 2012). Around one-third of cancer patients in Western cultures report continuous clinically significant discomfort, such as depression and anxiety, post-traumatic stress reactions, worries of cancer recurrence, and adjustment disorders, which may exacerbate over time (Zabora et al., 2001; Stein et al., 2008). In the 1970s, psycho-oncology came into existence to decrease these psychological symptoms and provide psychosocial assistance to cancer patients, caregivers, and families. Psycho-oncology is a cross-disciplinary and collaborative sub-specialty of oncology that focuses on the psychological, behavioral, ethical, and social aspects of cancer in clinical settings. The discipline also offers research and clinical materials to health professionals (Holland, 2002, 2018). Psychosocial rehabilitation and psychotherapeutic interventions in oncology have been shown to generally benefit and reduce the severity of psychiatric symptoms and somatic symptoms (Johannsen et al., 2013; Zainal et al., 2013).

Psycho-oncology focuses on the cancer patient to improve their well-being, quality of life, and return to work (Hunter et al., 2017a,b) and focuses on possible survival and illness behavior (Galway et al., 2012; Spiegel, 2012; Barrera and Spiege, 2014). The supportive-expressive group psychotherapy is one of the most empirically supported therapies for relieving cancer patients’ distress (Classen et al., 2008), cognitive-existential therapy (Kissane et al., 2003), and cognitive-behavioral (Johnson et al., 2016). Some other interventions are meaning-centered psychotherapy, mindfulness stress reduction, and mindfulness (LeMay and Wilson, 2008; Piet et al., 2012; Breitbart et al., 2015; Rouleau et al., 2015; Carlson, 2016; Okuyama et al., 2017; van der Spek et al., 2017).

The bibliometric analysis of published articles provides insights into the research landscape, research gaps, and future direction of a research field (Blakeman, 2018; Tang et al., 2018; Yeung et al., 2018). In this study, we performed bibliometric analysis to examine the published scientific literature and trend analysis of psycho-oncology-related research. We also identified socio-economic factors affecting research productivity in psycho-oncology.

## Methodology

### Study Design and Retrieved Database

A bibliometric and visualization study was conducted. In this study, the Scopus^®^ database (Elsevier, Amsterdam, Netherlands) was utilized. The used database was accessed through the online library portal of the University of the Philippines Manila, on 20 April 2022 (step 1-initial search), and Southeast University, Nanjing, on 28 May 2022 (step 2-updated search). The Scopus^®^ database was selected; (a) it is the world’s largest abstract and citation database for scientific literature (Guz and Rushchitsky, 2009), (b) it includes a more expanded spectrum of journals (Falagas et al., 2008).

### Used Keywords and Data Extraction

**FIGURE 1:**
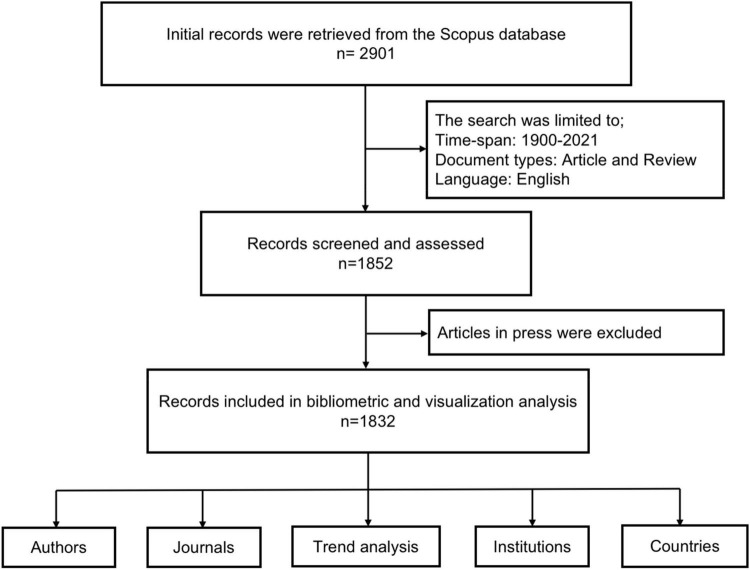
Flow diagram of the included publications.

A comprehensive search of the published literature was conducted. Published literature on psycho-oncology was screened and reviewed. The search keywords were discussed and reviewed by the authors to conduct a comprehensive search operation. Thus, the following potential search keywords were selected and entered in the database: “Psycho-oncology” OR “Psycho oncology” OR “Psychiatric oncology” OR “Psychosocial oncology” in the title, abstract, and keywords category. We limited the search to documents published in English and document types (article and review). Early access documents and publications in the year 2022 were also excluded as shown in Figure 1. A two-step search was conducted to validate all the results and data extraction. The authors extracted the following information; publication title, year of publication, publishing language, author name, journal, document type, research area, funding source, institution, and country of origin. The data were downloaded in both comma-separated values (CSV) and BibTeX format. The journal impact factor (IF) was obtained from the Journal Citation Reports 2020, released in June 2021 by Clarivate Analytics.

### Data Analysis

To analyze, characterize, and map the psycho-oncology-related research, the downloaded dataset was exported into RStudio (Biblioshiny package). The key bibliometric indicators were examined that included annual scientific production, most relevant authors and journals, leading institutions, countries or regions collaboration, keywords analysis, and trend analysis of topics. However, the leading funding sources, most studied subject areas, and countries or regions that participated in psycho-oncology-related research were plotted in Microsoft Excel. The values were presented in frequency (n) and percentage (%).

### Lotka’s and Bradford’s law

Furthermore, the author’s productivity was examined by using Lotka’s law. Lotka’s law demonstrates the number of authors against the number of contributions (papers) made by each author (Lotka, 1926). Lotka’s law is expressed in mathematical terms by the following formula:


$$\text{A}\,\left(\text{n}\right)=\frac{\text{A}\,\left(1\right)}{{\text{n}}^{2}}$$

In the above equation, A (*n*) is the number of authors publishing *n* papers and A1 is the number of authors publishing a single paper.

In addition, to find out the core journals in psycho-oncology field, Bradford’s law was used (Bradford, 1934). Bradford’s law distributes the journals into three zones [zone 1 (core journals), zone 2, and zone 3] with a similar number of papers and an increasing number of journals.

### Statistical Analysis

Spearman’s rank-order correlation determined correlations between country-specific characteristics with the different bibliometric indices. The information on the population, gross domestic product (GDP), GDP per capita, research and development expenditure (%GDP), physician-to-population ratio, and researcher-to-population ratio were obtained from the World Bank. The Spearman’s correlation coefficient (ρ) was considered significant if the *p*-value was less than 0.05. The statistical analysis was done using GraphPad Prism software version 7 (GraphPad Software, San Diego, CA).

### Ethical Consideration

Ethical consideration was not required for the current study because no animal and human subjects were involved. All the data used in this study are available publicly.

## Results

The initial search retrieved 2,906 documents, of which only 1,832 documents were analyzed and characterized in the final analysis. The included documents were authored by 7,363 authors (4.02 authors per document) and published in 490 journals. In total, 1,511 (82.48%) documents were published as articles and 321 (17.52%) as reviews. As presented in Table 1, the authors collaboration index in psycho-oncology research was found to be 4.38.

**TABLE 1:** Main information about the included documents in the final analysis.

Description	Results
Main information	
Time-span	1980–2021
Documents (records)	1,832
Journals	490
Institutions	6,382
Countries/regions	74
Average years from publication	7.7
Average citations per documents	17.7
Average citations per year per document	2.003
References	6,9946
Document types	
Article	1,511
Review	321
Document contents	
KeyWords Plus	5,467
Author’s keywords	2,919
Authors	
Authors	7,363
Author appearances	10,286
Authors of single-authored documents	141
Authors of multi-authored documents	7,222
Authors collaboration	
Single-authored documents	184
Documents per author	0.249
Authors per document	4.02
Co-authors per document	5.61
Collaboration index	4.38

### Annual Scientific Production

The included documents were published between 1980 and 2021. There is a general increase in research output through the years, especially in the last 5 years. The most productive year in terms of publications was 2021 (*n* = 365), and 2020 (*n* = 251), as shown in Table 2. In addition, the mean total citations per document and per year were calculated. The documents published in 2014 received the highest number of mean total citations per year (Table 2). The annual scientific growth rate was found to be 13.9%.

**TABLE 2:** Annual scientific production and mean citations.

Year	Number	Mean total citations per document	Mean total citations per year	Citable years
1980	2	10.50	0.25	42
1981	2	10.50	0.26	41
1982	0	0.00	0.00	0
1983	2	8.50	0.22	39
1984	1	17.00	0.45	38
1985	3	3.67	0.10	37
1986	1	77.00	2.14	36
1987	3	69.00	1.97	35
1988	2	1.00	0.03	34
1989	4	9.75	0.30	33
1990	2	15.50	0.48	32
1991	4	36.50	1.18	31
1992	6	30.33	1.01	30
1993	10	8.00	0.28	29
1994	16	13.13	0.47	28
1995	13	31.62	1.17	27
1996	13	24.15	0.93	26
1997	10	22.50	0.90	25
1998	14	46.00	1.92	24
1999	23	37.43	1.63	23
2000	15	53.47	2.43	22
2001	14	40.71	1.94	21
2002	31	48.55	2.43	20
2003	16	54.19	2.85	19
2004	28	38.46	2.14	18
2005	25	41.68	2.45	17
2006	22	37.00	2.31	16
2007	20	56.35	3.76	15
2008	28	28.25	2.02	14
2009	34	44.82	3.45	13
2010	44	25.32	2.11	12
2011	60	22.78	2.07	11
2012	69	29.49	2.95	10
2013	78	27.26	3.03	9
2014	80	32.24	4.03	8
2015	69	26.03	3.72	7
2016	80	17.19	2.86	6
2017	104	15.44	3.09	5
2018	127	11.69	2.92	4
2019	141	9.84	3.28	3
2020	251	5.00	2.50	2
2021	365	1.84	1.84	1

### Most Relevant Authors

**FIGURE 2:**
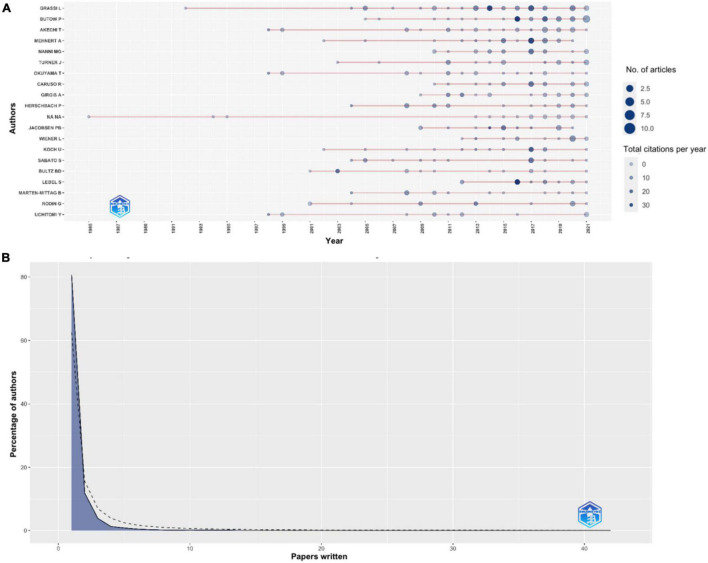
**(A)** Top authors’ production over time (1985–2021) in psycho-oncology research, **(B)** author productivity through Lotka’s law. The blue area corresponds to the empirical or observed data, whereas the dotted line represents plotted or theoretical relationship.

As shown in Table 1, a total of 7,363 were involved, and appeared to be 10,286 times. In total, only 43 authors published at least 10 documents. The most relevant leading author in terms of publications was Luigi Grassi (Grassi L) from the Department of Biomedical and Specialty Surgical Sciences, Institute of Psychiatry, University of Ferrara, Ferrara, Italy with 42 publications, followed by Phyllis Butow (Butow P) from the Psycho-Oncology Co-operative Research Group, School of Psychology, The University of Sydney, Sydney, Australia (*n* = 31), and Tatsuo Akechi (Akechi T) from the Department of Psychiatry and Cognitive-Behavioral Medicine, Nagoya City University Graduate School of Medical Sciences, Nagoya, Japan (*n* = 29), as shown in Table 3. As shown in Figure 2A, Butow P published the highest number of articles in 2021 (*n* = 10). The author productivity through Lotka’s law was calculated as shown in Figure 2B. As shown in Figure 2B and Table 4, in terms of scientific productivity (papers written) the number of authors declined as the number of papers written increased. Moreover, the paper titled “Cancer distress screening. Needs, models, and methods” published in Journal of Psychosomatic Research in 2003 was the most cited article with 279 citations (13.95 citations per year; Carlson and Bultz, 2003). This review discusses various screening instruments and screening models that could be widely adopted by psychosocial oncology programs.

**TABLE 3:** Most relevant authors with at least 15 publications.

Authors	Articles	Articles Fractionalized
Grassi L	42	10.71
Butow P	31	5.77
Akechi T	29	3.88
Mehnert A	22	4.30
Nanni MG	19	2.94
Turner J	19	4.51
Okuyama T	18	2.43
Caruso R	17	2.78
Girgis A	17	3.13
Herschbach P	16	1.98
Na Na	16	16.00
Jacobsen PB	15	3.66
Wiener L	15	2.41

**TABLE 4:** Lotka’s law statistics of papers written by authors in psycho-oncology research between 1980 and 2021.

Papers written	Number of authors	Proportion of authors
1	5,942	0.807
2	874	0.119
3	284	0.039
4	95	0.013
5	59	0.008
6	32	0.004
7	18	0.002
8	10	0.001
9	6	0.001
10	11	0.001
11	6	0.001
12	6	0.001
13	5	0.001
14	2	0
15	2	0
16	2	0
17	2	0
18	1	0
19	2	0
22	1	0
29	1	0
31	1	0
42	1	0

### Most Relevant Journals

**FIGURE 3:**
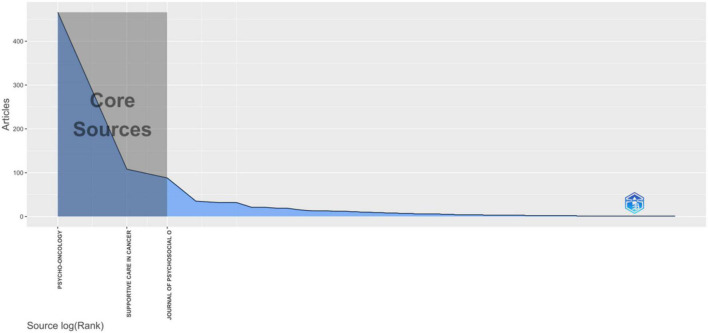
Journals (Sources) clustering through Bradford’s law.

As shown in Table 5, the most relevant and dominant journal in terms of publications was Psycho-Oncology (*n* = 466, 25.44%), followed by Supportive Care in Cancer (*n* = 108, 5.90%), and Journal of Psychosocial Oncology (*n* = 88, 4.80%). However, the most local cited journals were Psycho-Oncology, Journal of Clinical Oncology, and Cancer. Furthermore, the Bradford’s law was applied to assess the core journals in the field of psycho-oncology (Bradford, 1934). As shown in Figure 3, the core journals in the field of psycho-oncology were Psycho-Oncology, Supportive Care in Cancer, and Journal of Psychosocial Oncology. The document sources (journals) were divided in to three zones, zone 1 had 3 journals, zone 2 had 59, and zone 3 had 428, as shown in Table 6.

**TABLE 5:** Most relevant journals in psycho-oncology research.

Journals	Articles	IF 2020 (5-year)	Current publisher
Psycho-Oncology	466	3.894 (4.578)	Wiley111 River St, Hoboken 07030-5774, NJ
Supportive Care in Cancer	108	3.603 (3.958)	Springer, One New York Plaza, Suite 4600, New York, NY 10004, United States
Journal of Psychosocial Oncology	88	2.029 (2.111)	Routledge Journals, Taylor & Francis Ltd., 2-4 Park Square, Milton Park, Abingdon OX14 4RN, Oxon, England
Cancer	35	6.86 (7.921)	Wiley111 River St, Hoboken 07030-5774, NJ
European Journal of Cancer Care	32	2.52 (2.931)	Wiley111 River St, Hoboken 07030-5774, NJ
Palliative & Supportive Care	32	2.257 (2.541)	Cambridge Univ Press, 32 Avenue of The Americas, New York, NY 10013-2473
BMC Cancer	21	4.43 (4.372)	BMC, Campus, 4 Crinan St, London N1 9XW, England
Japanese Journal of Clinical Oncology	21	3.019 (2.847)	Oxford Univ Press, Great Clarendon St, Oxford OX2 6DP, England
Frontiers in Psychology	19	2.988 (3.618)	Frontiers Media SA, Avenue DU Tribunal Federal 34, Lausanne CH-1015, Switzerland
Journal of Pain and Symptom Management	19	3.612 (4.556)	Elsevier Science Inc., STE 800, 230 Park Ave, New York, NY 10169
Cancer Nursing	16	2.592 (2.98)	Lippincott Williams & Wilkins, Two Commerce SQ, 2001 Market St, Philadelphia, PA 19103

**TABLE 6:** Distribution of the journals (sources) and corresponding articles in three zones.

Zone	No. of journals	No. of articles	Percentage
1	3	662	36.14
2	59	567	30.95
3	428	603	32.91
Total	490	1,832	100

### Leading Institutions

**FIGURE 4:**
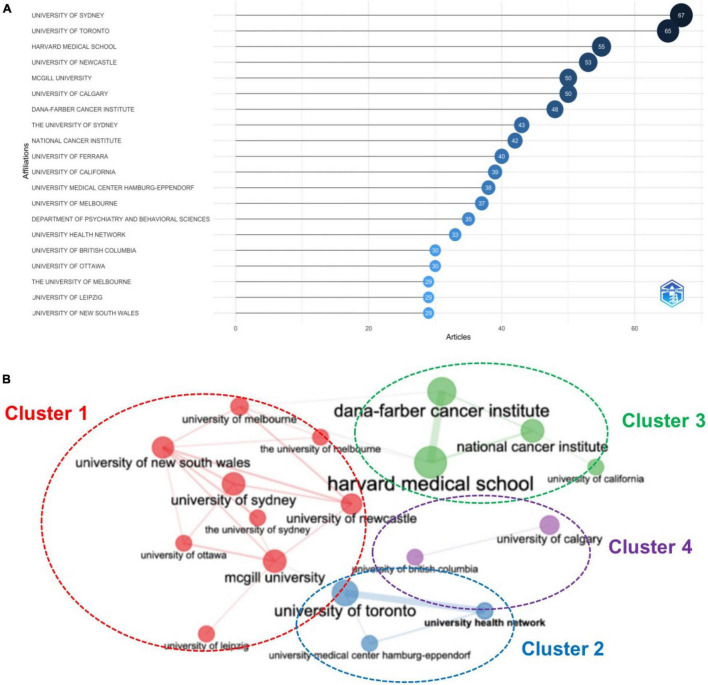
**(A)** Top 20 leading institutions in psycho-oncology research between 1980 and 2021, **(B)** Institutions collaboration network.

The leading institutions in psycho-oncology research were the University of Sydney (*n* = 67, 3.66%), followed by the University of Toronto (*n* = 65, 3.55%), and the Harvard Medical School (*n* = 55, 3.00%), as shown in Figure 4A. As presented in Figure 4B, the top leading institutions were plotted in to four clusters, and each color designates different cluster. The University of Toronto had the strongest collaboration with the University Health Network, while the Harvard Medical School had the highest collaboration with the DANA-Farber Cancer Institute.

### Top Leading Countries or Regions in Psycho-Oncology Research

**FIGURE 5:**
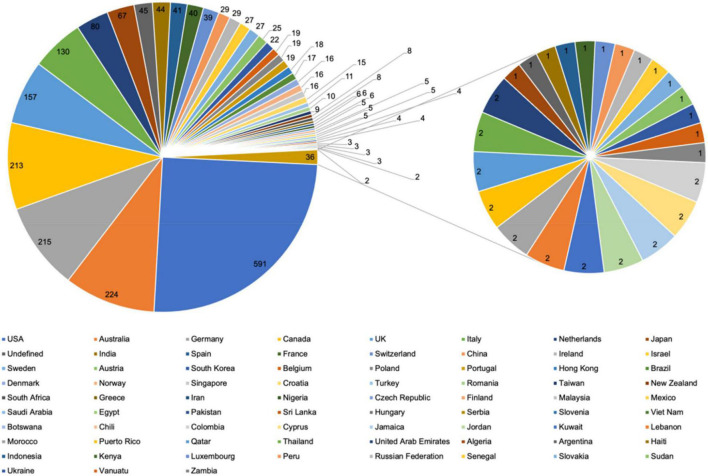
Countries or regions participated in psycho-oncology research between 1980 and 2021.

A total of 74 countries or regions participated in the included documents. As shown in Figure 5, the highly contributing country was the United States, followed by Australia, Germany, Canada, and the United Kingdom. As shown in Table 7, the United States was the leading country in terms of single country corresponding author’s publications (*n* = 392), followed by Germany (*n* = 150), and Australia (*n* = 128), while in terms of multiple country corresponding author’s publications Australia was the top ranked country (*n* = 44), followed by the United States (*n* = 37), and Italy (*n* = 36).

**TABLE 7:** Top 20 leading countries/regions in psycho-oncology research between 1980 and 2021 based on the corresponding author’s country.

Countries/Regions	Articles	Single country publications	Multiple country publications	Multiple country publications ratio
United States	429	392	37	0.0862
Germany	178	150	28	0.1573
Australia	172	128	44	0.2558
Canada	156	127	29	0.1859
Italy	110	74	36	0.3273
United Kingdom	86	74	12	0.1395
Japan	61	59	2	0.0328
Netherlands	49	39	10	0.2041
India	36	28	8	0.2222
China	30	24	6	0.2
France	25	20	5	0.2
Ireland	22	14	8	0.3636
Spain	22	17	5	0.2273
Switzerland	22	10	12	0.5455
Korea	20	18	2	0.1
Sweden	19	15	4	0.2105
Israel	18	15	3	0.1667
Singapore	15	10	5	0.3333
Croatia	13	12	1	0.0769
Poland	13	13	0	0

### Countries or Regions Collaboration in Psycho-Oncology Research

**FIGURE 6:**
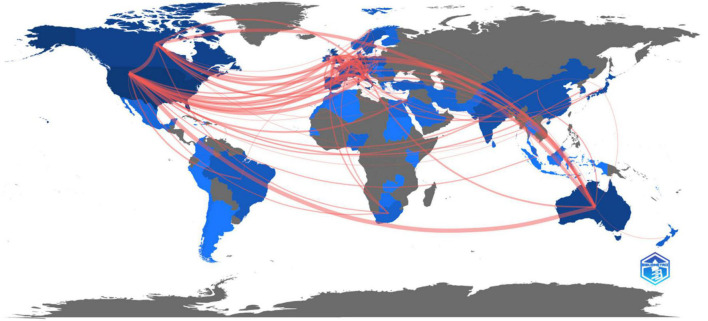
Collaboration world map of countries or regions involved in psycho-oncology research between 1980 and 2021.

As shown in Figure 6, the United States had the strongest collaboration with Australia in 38 publications, followed by the United States and Canada (*n* = 36), Australia and Canada (*n* = 23), the United States and Italy (*n* = 23), and Canada and the United Kingdom (*n* = 21).

### Leading Funding Agencies

**FIGURE 7:**
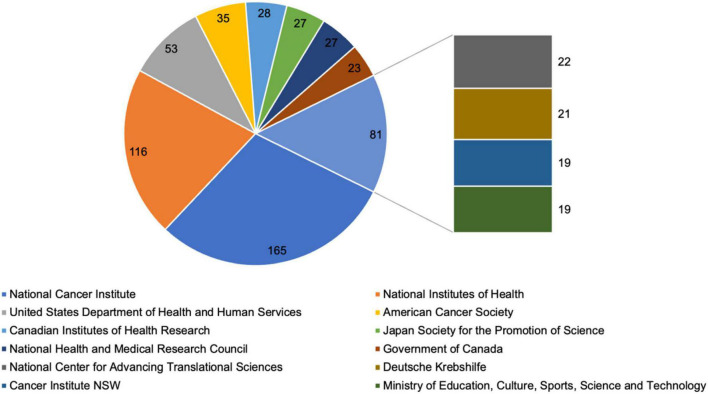
Leading funding agencies in psycho-oncology research between 1980 and 2021 with at least 15 funded studies.

As shown in Figure 7, the leading funding agency in psycho-oncology was the National Cancer Institute (*n* = 165), followed by the National Institutes of Health (NIH; *n* = 116), and the United States Department of Health and Human Services (HHS; *n* = 153).

### Most Studied Subject Areas

**FIGURE 8:**
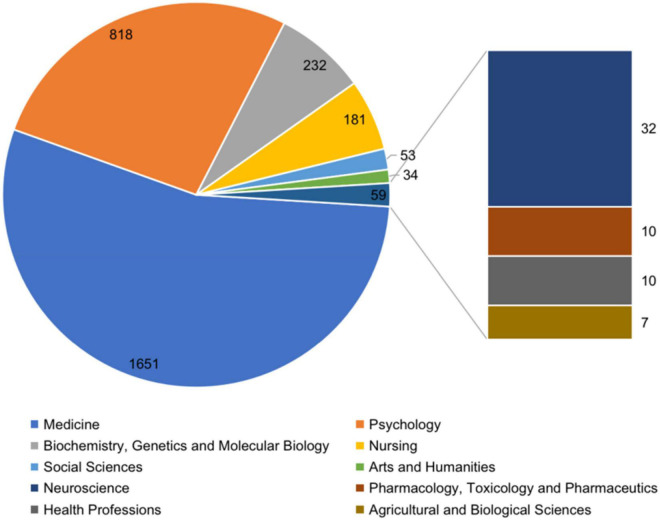
Most studied subject areas in psycho-oncology between 1980 and 2021.

The subject areas that covers psycho-oncology research are mainly on Medicine (*n* = 1,651), Psychology (*n* = 818) and Biochemistry, Genetics and Molecular Biology (*n* = 232), Nursing (*n* = 181), and Social Sciences (*n* = 53), as shown in Figure 8.

### Keywords Analysis

**FIGURE 9:**
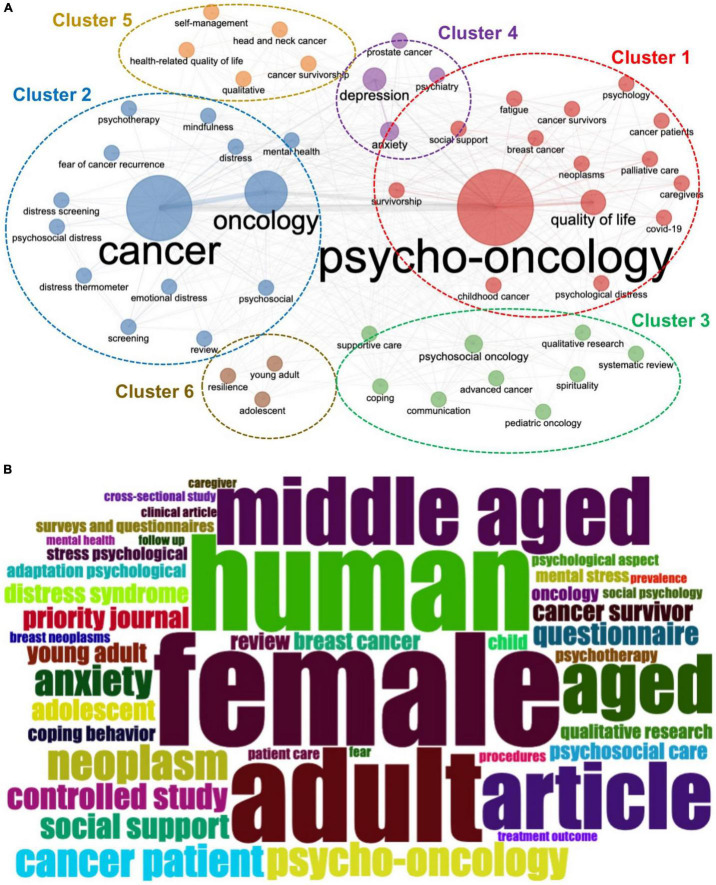
**(A)** Frequently used author’s keywords, **(B)** KeyWords Plus WordCloud map.

As shown in Figure 9A, the most frequently used author’s keywords were plotted into six clusters. The top ten most widely used keywords were psycho-oncology, cancer, oncology, quality of life, depression, anxiety, psychosocial oncology, distress, survivorship, and breast cancer. The most appeared KeyWords Plus were female, human, adult, male, humans, middle aged, article, aged, quality of life, and neoplasms as shown in Figure 9B.

### Trend Topics

**FIGURE 10:**
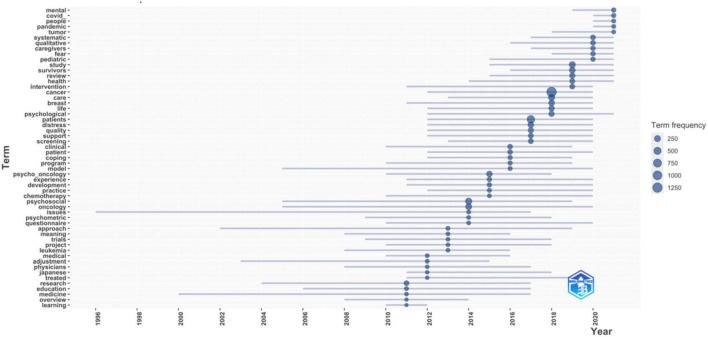
Trend topics in psycho-oncology in the last 10 years (2011–2021).

The trend topics analysis was performed based on publication title during the last decade as shown in Figure 10. The recent trend topics in psycho-oncology were related to mental health, COVID-19 infection in humans, people, pandemic, and tumor. In the last 10 years, the most focused trend topic was cancer, followed by patients, and psychosocial.

### Socio-Economic Factors for Psycho-Oncology Research Productivity

Lastly, we determined the socio-economic factors that may affect research productivity on psycho-oncology (Table 8). International collaboration showed the strongest correlation with the bibliometric indices (total publications: *r* = 0.920, *p* < 0.001; citations: *r* = 0.873, *p* < 0.001; h-index = 0.901, *p* < 0.001). Research and development expenditure (total publications: *r* = 0.690, *p* < 0.001; citations: *r* = 0.709, *p* < 0.001; h-index = 0.691, *p* < 0.001) and GDP (total publications: *r* = 0.730, *p* < 0.001; citations: *r* = 0.633, *p* < 0.001; h-index = 0.692, *p* < 0.001) also showed a significant positive correlation. Moreover, GDP per capita, researcher per million people, and physicians per population also exhibited a positive relationship. In addition, specific characteristics of the country or region involved in psycho-oncology research is presented in Supplementary Table 1.

**TABLE 8:** Correlation analysis between country or region-specific characteristics and bibliometric indices of psycho-oncology research.

Variables	Bibliometric Index	Spearman *R*	*P*-value
GDP	Total publications	0.730	<0.001
	H-index	0.692	<0.001
	Total citations	0.633	<0.001
GDP per capita	Total publications	0.591	<0.001
	H-index	0.581	<0.001
	Total citations	0.590	<0.001
Research and development expenditure (% of GDP)	Total publications	0.690	<0.001
	H-index	0.691	<0.001
	Total citations	0.709	<0.001
Researchers in R&D (per million people)	Total publications	0.572	<0.001
	H-index	0.593	<0.001
	Total citations	0.598	<0.001
Physicians per 1,000 population	Total publications	0.466	<0.001
	H-index	0.450	<0.001
	Total citations	0.453	<0.001
International collaboration	Total publications	0.920	<0.001
	H-index	0.901	<0.001
	Total citations	0.873	<0.001

## Discussion

Focused bibliometric studies are critical for providing key bibliometric indices such as most prolific authors and journals, top-ranked institutions and countries, scientific production over time and present the trend analysis of research in a particular field or research area (Donthu et al., 2021).

Although some previously published studies used a bibliometric approach to examine psycho-oncology-related specific domains (Fox et al., 2021). However, to the best of our knowledge, there is a lack of a comprehensive bibliometric analysis of psycho-oncology-related publications indexed in the Scopus^®^ database from inception up to 31 December 2021. Thus, this study allowed us to acquire information about the developments and research trends in psycho-oncology over the last four decades.

Generally, an increase in research productivity in psycho-oncology over the years was observed. In total, 1.20% of the publications were published between 1980 and 1990, 6.77% between 1991 and 2000, 14.30% between 2001 and 2010, and 77.73% between 2011 and 2021. Overall, less than 23% of the included publications were published between 1980 and 2010. However, the overall annual scientific growth rate was found to be 13.09%. The above statistics show that, in recent times, much more research was conducted in psycho-oncology which reflects the importance and improvement of psycho-oncology-related therapies.

Publications, journals, and books that reflect a field’s body of knowledge are indicators of advancement. The most relevant and top-ranked journal in terms of publications in psycho-oncology was “Psycho-Oncology.” According to the Journal Citation Reports released in 2021, “Psycho-Oncology” received a 3.894 IF and ranked Q1 (16/77 category ranking) and Q3 (136/242 category ranking) in psychology and oncology, respectively. The IF of the core journals (based on Bradford’s law) ranges from 2.029 (Journal of Psychosocial Oncology) to 3.894 (Psycho-Oncology).

Psycho-Oncology was launched in 1992 and is the official journal of the “American Psychosocial Oncology Society” (APOS) and the “British Psycho-Oncology Society” (BPOS). The journal covers psychological, social, behavioral, and ethical aspects of cancer. This year the journal celebrating its 30th anniversary.

The main subject areas that published psycho-oncology research are medicine, psychology, and biochemistry, genetics, and molecular biology. Notably, subject areas include social sciences, arts and humanities, computer science, and engineering. This highlights the multidisciplinary nature of the research field. The keyword analysis revealed topics on mental health issues such as depression and anxiety. Moreover, growing research trends between 1980 and 2000 in psycho-oncology-related research were support and psychiatric nursing, clinical trials, and imipramine. However, between 2001 and 2010, the topics related to psychological aspects, adaptive behavior and therapy, and cancer were more emphasized and developed. Trend topics over the last decade (2011–2020) included neoplasm, human(s), female, age, psychology, and social support. Psycho-oncology developed as a sub-specialty of oncology contributing to cancer patients’ care (Holland, 2002).

The United States is the leading country in psycho-oncology research productivity and is home to most of the top institutions and funding agencies in this field. Previously published bibliometric studies in different research fields also reported the highest contribution from the United States, such as congenital cataracts (Idriss et al., 2021), exosomes (Shi et al., 2021), fascioliasis (Ahmad et al., 2021c), hepatitis (Ahmad et al., 2021e,f), myocardial infarction (Zhou et al., 2018), pediatric trauma (Ahmad et al., 2021b), psychosomatic (Shah et al., 2021), tuberculosis (Nafade et al., 2018), and vaccines (Zhang et al., 2019; Ahmad et al., 2021a,d).

Several factors influence the country’s research productivity. Our results showed that international collaboration is strongly associated with all the bibliometric indices. This is consistent with the literature showing that cooperation and collaboration increase research productivity (Tantengco, 2021; Ornos and Tantengco, 2022). Furthermore, GDP and research expenditure are strongly correlated with bibliometric indices of psycho-oncology research. International collaboration in psycho-oncology-related research is very strong among developed countries.

### Limitations

This study has several limitations. Firstly, all the data used in this study were retrieved from a single database. The use of other databases such as Google Scholar, PubMed, and Web of Science would yield more publications on psycho-oncology-related research. Secondly, the search was performed with some specific restrictions which limited the number of publications.

## Conclusion

This study presents a bench of evidence that might have several applications for clinicians, researchers, and policymakers working in the field of psycho-oncology. A significant increase in psycho-oncology-related publications has been observed in the last 5 years. The annual scientific growth rate was found to be 13.9%. The most highly contributing and collaborative country was the United States. The top leading author and institution were Grassi L and the University of Sydney, respectively. Lotka’s law found that the number of authors declined as the number of papers written increased. The overall authors collaboration index was 4.38. The top-ranked core journal was Psycho-Oncology. The research hotspots include mental health conditions and interventions in cancer patients. International collaboration and research expenditure are strongly associated with psycho-oncology research productivity. Researchers’ collaboration, which is visible among developed countries, should be extended to low-income countries in order to expand psycho-oncology-related research and understanding.

## Data Availability Statement

The original contributions presented in the study are included in the article/Supplementary Material, further inquiries can be directed to the corresponding authors.

## Author Contributions

TA: conceptualization and study design. TA and EO: methodology, data curation, software, and formal analysis. TA: analysis, visualization, and preparation of the first draft. TA, EO, SA, RA-W, IM, SS, BA-O, MB, and KT: data assessment and validation, manuscript writing, review, and editing. TA: project administration and supervision. All authors read and approved the current version for publication.

## Conflict of Interest

The authors declare that the research was conducted in the absence of any commercial or financial relationships that could be construed as a potential conflict of interest.

## Publisher’s Note

All claims expressed in this article are solely those of the authors and do not necessarily represent those of their affiliated organizations, or those of the publisher, the editors and the reviewers. Any product that may be evaluated in this article, or claim that may be made by its manufacturer, is not guaranteed or endorsed by the publisher.
