# Supplementary material for: Global Research Mapping of Psycho-Oncology Between 1980 and 2021: A Bibliometric Analysis
Source: Front Psychol. 2022 Jul 13;13:947669. doi: 10.3389/fpsyg.2022.947669 (PMC9326365; doi:10.3389/fpsyg.2022.947669)
Supplement: Supplementary file 1 [file Table_1.DOCX]

**SUPPLMENTARY TABLE**

Supplementary Table 1: Specific characteristics of country or region involved in psycho-oncology research

| **Country or Region** | **Total publications** | **H-Index** | **Total citations** | **GDP** | **GDP per capita** | **Research and development expenditure (% of GDP)** | **Researchers in R&D (per million people)** | **Physicians per 1000 population** | **International collaboration (Links)** | **International collaboration (Total link strength)** |
| --- | --- | --- | --- | --- | --- | --- | --- | --- | --- | --- |
| USA | 592 | 55 | 12221 | 2.09E+13 | 63413.51 | 2.83283 | 4412.435 | 2.612 | 35 | 144 |
| Germany | 215 | 33 | 3834 | 3.85E+12 | 46208.43 | 3.13267 | 5211.874 | 4.2488 | 24 | 55 |
| Australia | 224 | 32 | 3735 | 1.33E+12 | 51692.84 | 1.87466 | 4532.401 | 3.6778 | 24 | 87 |
| Canada | 213 | 35 | 4948 | 1.64E+12 | 43258.18 | 1.54266 | 4325.645 | 2.6102 | 24 | 78 |
| UK | 157 | 29 | 3669 | 2.71E+12 | 41124.53 | 1.70274 | 4603.31 | 2.8117 | 29 | 73 |
| Italy | 130 | 26 | 2599 | 1.89E+12 | 31714.22 | 1.39181 | 2306.766 | 3.9774 | 25 | 55 |
| Netherlands | 80 | 25 | 2416 | 9.14E+11 | 52397.12 | 2.16374 | 5604.541 | 3.6054 | 21 | 39 |
| Japan | 67 | 19 | 1210 | 4.98E+12 | 40193.25 | 3.27512 | 5331.15 | 2.4115 | 14 | 7 |
| France | 40 | 12 | 562 | 2.63E+12 | 39030.36 | 2.19294 | 4715.319 | 3.2672 | 22 | 19 |
| Spain | 41 | 17 | 909 | 1.28E+12 | 27063.19 | 1.24323 | 3000.887 | 3.8723 | 21 | 19 |
| India | 44 | 11 | 361 | 2.62E+12 | 1927.708 | 0.65282 | 252.7039 | 0.8571 | 9 | 12 |
| Switzerland | 39 | 13 | 732 | 7.52E+11 | 87097.04 | 3.36798 | 5450.432 | 4.2957 | 16 | 29 |
| China | 29 | 5 | 104 | 1.47E+13 | 10434.78 | 2.14058 | 1307.121 | 1.9798 | 5 | 7 |
| Ireland | 29 | 9 | 206 | 4.26E+11 | 85267.76 | 1.14647 | 5243.126 | 3.3125 | 14 | 12 |
| Sweden | 27 | 10 | 322 | 5.41E+11 | 52274.41 | 3.31278 | 7536.475 | 3.984 | 14 | 12 |
| Israel | 27 | 13 | 525 | 4.02E+11 | 44168.94 | 4.94079 | 8250.48 | 4.6249 | 11 | 10 |
| Austria | 25 | 9 | 285 | 4.31E+11 | 48586.8 | 3.2105 | 5733.076 | 5.1697 | 14 | 17 |
| Korea, Rep. | 22 | 8 | 177 | 1.63E+12 | 31631.47 | 4.52753 | 7980.396 | 2.3608 | 1 | 4 |
| Brazil | 17 | 6 | 141 | 1.44E+12 | 6796.845 | 1.16038 | 887.6784 | 2.1643 | 10 | 7 |
| Hong Kong, China | 18 | 5 | 95 | 3.47E+11 | 46323.86 | 0.8634 | 4026.599 |  | 9 | 9 |
| Poland | 19 | 8 | 138 | 5.94E+11 | 15720.99 | 1.20953 | 3106.12 | 2.3788 | 13 | 5 |
| Belgium | 19 | 9 | 518 | 5.15E+11 | 45159.35 | 2.76578 | 5023.263 | 3.0709 | 18 | 13 |
| Croatia | 15 | 3 | 43 | 5.6E+10 | 14134.16 | 0.97192 | 1921.131 | 2.9996 | 3 | 2 |
| Portugal | 19 | 12 | 441 | 2.31E+11 | 22176.3 | 1.34753 | 4537.532 | 5.124 | 17 | 12 |
| Singapore | 16 | 8 | 216 | 3.4E+11 | 59797.75 | 1.92465 | 6802.537 | 2.2936 | 3 | 6 |
| Denmark | 16 | 11 | 610 | 3.56E+11 | 61063.32 | 3.03292 | 8065.887 | 4.0099 | 13 | 8 |
| Norway | 16 | 10 | 313 | 3.63E+11 | 67389.91 | 2.07264 | 6466.7 | 2.9164 | 10 | 14 |
| Turkey | 11 | 6 | 107 | 7.2E+11 | 8536.433 | 0.95978 | 1379.412 | 1.8492 | 1 | 1 |
| Romania | 10 | 3 | 31 | 2.49E+11 | 12896.09 | 0.50077 | 882.4413 | 2.9807 | 5 | 2 |
| Taiwan | 9 | 5 | 117 | 6.68E+11 | 33004.00 | 3.49 |  |  | 2 | 3 |
| New Zealand | 8 | 5 | 158 | 2.11E+11 | 41441.47 | 1.34692 | 5529.522 | 3.5898 | 1 | 2 |
| South Africa | 8 | 5 | 155 | 3.02E+11 | 5655.868 | 0.83215 | 517.7219 | 0.9054 | 7 | 5 |
| Mexico | 5 | 2 | 5 | 1.08E+12 | 8329.271 | 0.31291 | 315.2625 | 2.3827 | 2 | 3 |
| Nigeria | 6 | 3 | 68 | 4.32E+11 | 2097.092 | 0.13223 | 38.78981 | 0.3806 | 8 | 4 |
| Czech Republic | 5 | 2 | 38 | 2.45E+11 | 22932.22 | 1.93017 | 3862.671 | 4.1208 | 10 | 2 |
| Greece | 6 | 3 | 29 | 1.89E+11 | 17622.54 | 1.17732 | 3482.717 | 5.4789 | 9 | 3 |
| Iran, Islamic Rep. | 6 | 2 | 32 | 1.92E+11 | 2422.481 | 0.83027 | 1474.914 | 1.5844 | 6 | 3 |
| Finland | 5 | 3 | 48 | 2.7E+11 | 48773.28 | 2.75572 | 6861.11 | 3.8118 | 4 | 3 |
| Malaysia | 5 | 2 | 15 | 3.37E+11 | 10412.35 | 1.04086 | 2184.722 | 1.5358 | 5 | 1 |
| Saudi Arabia | 5 | 2 | 13 | 7E+11 | 20110.32 | 0.81516 |  | 2.6117 | 3 | 3 |
| Egypt, Arab Rep. | 4 | 2 | 7 | 3.63E+11 | 3569.207 | 0.72388 | 686.7174 | 0.4521 | 0 | 0 |
| Pakistan | 4 | 2 | 6 | 2.64E+11 | 1188.86 | 0.23627 | 335.5807 | 0.9801 | 0 | 0 |
| Sri Lanka | 4 | 3 | 32 | 8.07E+10 | 3680.673 | 0.12758 | 106.3989 | 1.0041 | 0 | 0 |
| Hungary | 3 | 1 | 10 | 1.55E+11 | 15980.74 | 1.53337 | 3237.703 | 3.4075 | 0 | 0 |
| Serbia | 3 | 1 | 4 | 5.3E+10 | 7720.511 | 0.9197 | 2087.219 | 3.1131 | 0 | 0 |
| Slovenia | 3 | 3 | 202 | 5.36E+10 | 25517.33 | 1.95039 | 4854.568 | 3.0861 | 0 | 0 |
| Vietnam | 3 | 2 | 6 | 2.71E+11 | 2785.724 | 0.52674 | 707.7436 | 0.8281 | 0 | 0 |
| Botswana | 2 | 1 | 3 | 1.58E+10 | 6404.9 | 0.53728 | 185.2078 | 0.5269 | 0 | 0 |
| Chile | 2 | 2 | 28 | 2.53E+11 | 13231.7 | 0.35608 | 493.2954 | 2.5912 | 0 | 0 |
| Colombia | 2 | 1 | 9 | 2.71E+11 | 5334.556 | 0.23462 | 88.0191 | 2.1848 | 0 | 0 |
| Cyprus | 2 | 1 | 1 | 2.38E+10 | 26623.8 | 0.54783 | 1255.853 | 1.9509 | 0 | 0 |
| Jamaica | 2 | 1 | 4 | 1.38E+10 | 4664.53 | 0.06096 |  | 1.3061 | 0 | 0 |
| Jordan | 2 | 1 | 2 | 4.37E+10 | 4282.766 | 0.70781 | 595.963 | 2.3237 | 0 | 0 |
| Kuwait | 2 | 2 | 52 | 1.36E+11 | 24811.77 | 0.06238 | 513.8602 | 2.6463 | 0 | 0 |
| Lebanon | 2 | 2 | 20 | 3.34E+10 | 4649.548 |  |  | 2.1038 | 0 | 0 |
| Morocco | 2 | 2 | 31 | 1.13E+11 | 3009.249 | 0.71454 | 1073.54 | 0.7308 | 0 | 0 |
| Puerto Rico | 2 | 1 | 3 | 1.03E+11 | 32290.92 | 0.42883 | 348.3643 |  | 0 | 0 |
| Qatar | 2 | 1 | 7 | 1.46E+11 | 50124.39 | 0.50901 | 577.3485 | 2.4852 | 0 | 0 |
| Thailand | 2 | 1 | 4 | 5.02E+11 | 7186.874 | 1.00179 | 1350.343 | 0.805 | 0 | 0 |
| United Arab Emirates | 2 | 2 | 19 | 4.21E+11 | 36284.56 | 1.27836 | 2378.889 | 2.5278 | 0 | 0 |
| Algeria | 1 | 1 | 2 | 1.45E+11 | 3306.858 | 0.54297 | 819.3427 | 1.7193 | 0 | 0 |
| Argentina | 1 | 1 | 2 | 3.83E+11 | 8579.018 | 0.4936 | 1211.42 | 3.9901 | 0 | 0 |
| Haiti | 1 | 1 | 3 | 1.34E+10 | 1272.368 |  |  | 0.2343 | 0 | 0 |
| Indonesia | 1 | 1 | 2 | 1.06E+12 | 3869.588 | 0.22633 | 215.9936 | 0.4269 | 0 | 0 |
| Kenya | 1 | 1 | 23 | 9.88E+10 | 1878.581 | 0.78577 | 221.3859 | 0.1565 | 0 | 0 |
| Luxembourg | 1 | 0 | 0 | 7.33E+10 | 116014.6 | 1.21126 | 4941.704 | 3.009 | 0 | 0 |
| Peru | 1 | 0 | 0 | 2.02E+11 | 6126.875 | 0.12715 |  | 1.3048 | 0 | 0 |
| Russian Federation | 1 | 0 | 0 | 1.48E+12 | 10126.72 | 0.98275 | 2784.332 | 4.0139 | 0 | 0 |
| Senegal | 1 | 1 | 3 | 2.49E+10 | 1471.831 | 0.5772 | 564.3395 | 0.0691 | 0 | 0 |
| Slovak Republic | 1 | 1 | 4 | 1.05E+11 | 19266.51 | 0.83806 | 2995.958 | 3.4156 | 0 | 0 |
| Sudan | 1 | 1 | 3 | 2.61E+10 | 595.4678 | 0.29844 |  | 0.2618 | 0 | 0 |
| Ukraine | 1 | 0 | 0 | 1.56E+11 | 3726.927 | 0.47109 | 988.0766 | 2.9923 | 0 | 0 |
| Vanuatu | 1 | 1 | 4 | 8.55E+08 | 2870.089 |  |  | 0.1653 | 0 | 0 |
| Zambia | 1 | 1 | 13 | 1.93E+10 | 985.1324 | 0.27819 | 41.71683 | 0.0925 | 0 | 0 |
